# Pilot Study of ^64^CuCl_2_ for PET Imaging of Inflammation

**DOI:** 10.3390/molecules23020502

**Published:** 2018-02-24

**Authors:** Lei Jiang, Dongli Song, Hao Chen, Ao Zhang, Huoqiang Wang, Zhen Cheng

**Affiliations:** 1 Department of Nuclear Medicine, Shanghai Pulmonary Hospital, Tongji University School of Medicine, Shanghai 200433, China; leijiang1031@163.com; 2 Molecular Imaging Program at Stanford (MIPS), Department of Radiology and Bio-X Program, Canary Center at Stanford for Cancer Early Detection, Stanford University, Stanford, CA 94305-5484, USA; haoc@stanford.edu; 3 Institute of Clinical Science, Zhongshan Hospital, Fudan University, Shanghai 200032, China; songdongli37@126.com; 4 CAS Key Laboratory of Receptor Research, Synthetic Organic & Medicinal Chemistry Laboratory (SOMCL), Shanghai Institute of Materia Medica, Chinese Academy of Sciences, No. 555 Zuchong Road, Pudong New Area, Shanghai 201203, China; aozhang@simm.ac.cn

**Keywords:** ^64^CuCl_2_, copper transporter 1 (CTR1), PET, inflammation

## Abstract

Copper(II) ion (Cu^2+^) is the essential element for numerous pathophysiological processes in vivo. Copper transporter 1 (CTR1) is mainly responsible for maintaining Cu^2+^ accumulation in cells, which has been found to be over-expressed in inflammatory tissues. Therefore, we explored the potential application of ^64^CuCl_2_ for PET imaging of inflammation through targeting CTR1. The animal models of H_2_O_2_ induced muscle inflammation and lipopolysaccaharide induced lung inflammation were successfully established, then imaged by small animal PET (PET/CT) post-injection of ^64^CuCl_2_, and PET images were quantitatively analyzed. H&E and immunohistochemical (IHC) staining and western blot experiments were performed for evaluating CTR1 levels in the inflammatory and control tissues. Both inflammatory muscle and lungs can be clearly imaged by PET. PET image quantitative analysis revealed that the inflammatory muscle and lungs showed significantly higher ^64^Cu accumulation than the controls, respectively (*p* < 0.05). Furthermore, IHC staining and western blot analysis demonstrated that compared with the controls, CTR1 expression was increased in both the inflammatory muscle and lungs, which was consistent with the levels of ^64^Cu^2+^ accumulation in these tissues. ^64^CuCl_2_ can be used as a novel, simple, and highly promising PET tracer for CTR1 targeted imaging of inflammation.

## 1. Introduction

Copper(II) ion (Cu^2+^) is an essential element for many pathophysiological processes in living subjects, such as respiration, iron transport, oxidative stress protection, peptide hormone production, pigmentation, blood clotting, normal cell growth, and so on [1,2,3,4]. Although Cu^2+^ is not always abundant in the internal environment, Cu^2+^ homeostasis is tightly regulated through a delicate and complex network of influx copper transporter, efflux copper transporters (ATP7A and ATP7B), copper chaperons (ATOX1, Cox17, CCS), and other copper binding molecules [3,5]. The copper transporter 1 (CTR 1) protein is a high-affinity Cu^2+^ transporter, which mainly functions as regulation of copper accumulation in organisms with the range from yeast to mammals [6,7]. 

Importantly, CTR1 has been found to be over-expressed in Alzheimer′s disease and a variety of cancers. It then has been explored as a novel target for tumor imaging and therapy in recent years [8,9,10,11,12,13,14]. For example, ^64^CuCl_2_, as the substrate of CTR1, has been demonstrated as a potential positron emission tomography (PET) tracer for imaging animal models with melanoma, liver cancer, and prostate cancer [10,12,14,15]. Recently, Piccardo A et al. [16] showed that ^64^CuCl_2_-PET/CT shows a significantly higher detection rate in human prostate cancer than ^18^F-Choline-PET/CT. These studies have inspired us to explore more imaging applications of ^64^CuCl_2_-PET in nuclear medicine.

Inflammation is the response of the immune system that guards the body against several harmful stimuli in normal conditions. Inflammation is involved in various side effects such as gastrointestinal toxicity, mucositis, skin reactions, nervous system damage, pneumonitis, fibrosis, and so on. Therefore, early and accurate diagnosis of the inflammation is important for reducing the normal tissue injury and improving the therapeutic effect [17]. In the early 1980s, Milanino R et al. [18] and Conforti A et al. [19] found that acute and chronic inflammations featured with changes in the metabolism of Cu. Furthermore, Gomathy Narayanan I. et al. reported that CTR1 was significantly increased in Eales disease, which highlights the role of CTR1 in the pathogenesis of inflammation [20].

Therefore, in this work, we hypothesized that ^64^CuCl_2_-PET can be a potential tool for inflammation imaging and further investigated the applications of ^64^CuCl_2_ in CTR1 targeted PET imaging of inflammation. 

## 2. Results

### 2.1. Small Animal PET/CT of Inflammatory Muscle

**Figure 1:**
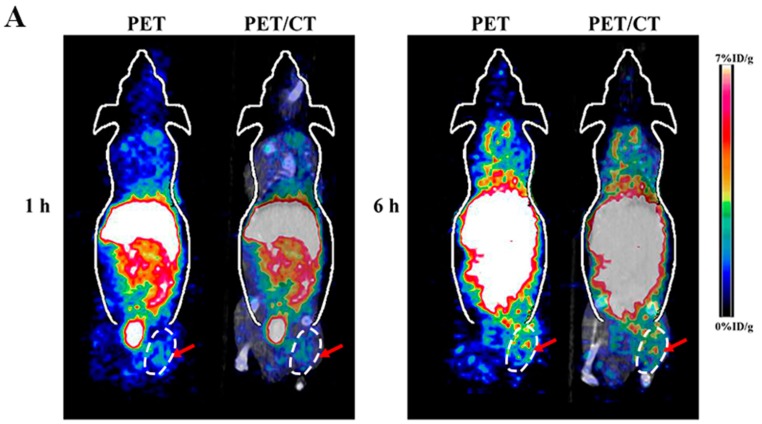

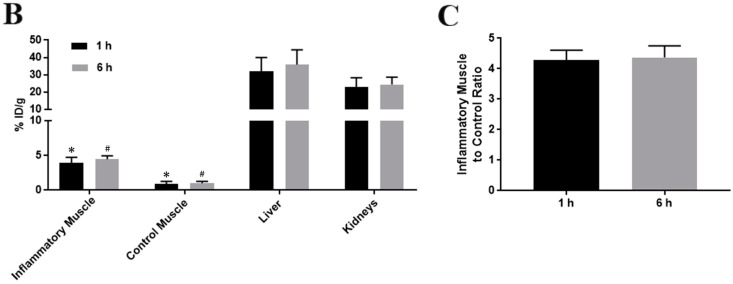
(**A**) Decay-corrected whole-body coronal small animal PET and PET/CT images of animal models of H_2_O_2_ induced muscle inflammation at 1 h and 6 h after intravenous injection of ^64^CuCl_2_, respectively (the inflammatory muscle tissues are indicated by arrows); (**B**) Small animal PET quantification analysis of inflammatory muscle, control muscle and major organs (liver and kidney) at 1 h and 6 h post-injection of radioactive ^64^Cu^2+^, respectively (*n* = 4) (* *p* = 0.021, ^#^ *p* = 0.036); (**C**) Small animal PET quantification analysis of ratio of inflammatory muscle to control muscle at 1 h and 6 h post-injection of radioactive copper, respectively (*n* = 4).

Representative coronal small animal PET and PET/CT images of animal models of H_2_O_2_ induced muscle inflammation at 1 h and 6 h after injection of ^64^CuCl_2_ were displayed in Figure 1A. The radioactivity accumulated in the inflammatory muscle of the right hind leg was observed at early time point of 1 h and following 6 h post-injection (p.i.). High liver and kidney accumulation were also observed, which verified the hepatobiliary and renal clearance route of radioactive ^64^Cu^2+^. Moreover, other normal organs and tissues showed relatively low radioactivity accumulation.

The quantification analysis (Figure 1B) demonstrated that radioactivity uptake of the inflammatory muscle was 3.90 ± 0.83%ID/g and 4.46 ± 0.49%ID/g at 1 and 6 h p.i., respectively. Meanwhile, the control muscle uptake of radioactivity was the background level, which was 0.91 ± 0.38%ID/g and 1.02 ± 0.27%ID/g at 1 h and 6 h p.i., respectively. There was obviously difference between the radioactivity accumulation of the inflammatory muscle and the control muscle (* *p* = 0.021, ^#^ *p* = 0.036). The average ratio of the inflammatory muscle to the control was 4.29 ± 0.32 and 4.37 ± 0.38 at 1 and 6 h p.i., respectively (Figure 1C). The liver and kidney accumulation of radioactive ^64^Cu^2+^ was 32.01 ± 7.94%ID/g and 22.83 ± 5.51%ID/g at 1 h p.i., respectively, and 35.87 ± 8.53%ID/g and 24.46 ± 4.19%ID/g at 6 h p.i., respectively, which was consistent with the results of PET/CT imaging (Figure 1B). 

### 2.2. Small Animal PET of Inflammatory Lungs

**Figure 2:**
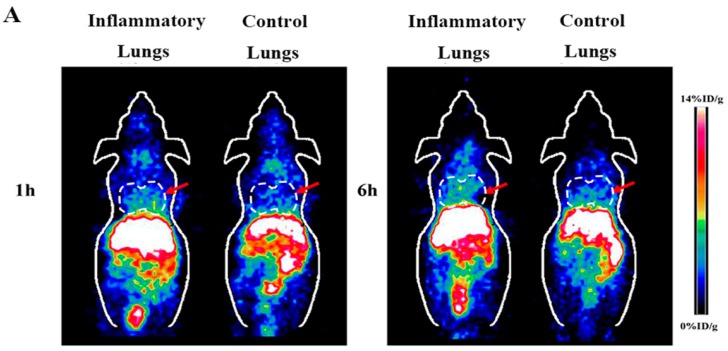

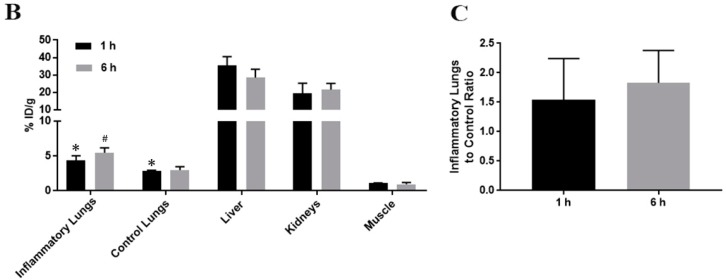
(**A**) Decay-corrected whole-body coronal small animal PET images of animal models of LPS induced lung inflammation at 1 h and 6 h after intravenous injection of ^64^CuCl_2_, respectively (The inflammatory lungs are indicated by arrows); (**B**) Small animal PET quantification analysis of inflammatory lungs, control lungs and major organs (liver, kidney and muscle) at 1 h and 6 h post-injection of radioactive copper, respectively (*n* = 4) (* *p* = 0.027, ^#^ *p* = 0.009); (**C**) Small animal PET quantification analysis of ratio of inflammatory lungs to control lungs at 1 h and 6 h post-injection of radioactive copper, respectively (*n* = 4).

Representative coronal small animal PET images of the animal models of lipopolysaccaharide (LPS) induced lung inflammation at the time point of 1 h and 6 h after injection of ^64^CuCl_2_ were displayed in Figure 2A. Compared with the control group, much more radioactivity was observed in the inflammatory lungs than in the lungs of normal mice. High liver and kidney accumulation were also observed, and other normal tissues and organs showed relatively low accumulation of ^64^Cu^2+^.

Further quantification analysis (Figure 2B) showed that ^64^Cu^2+^ uptake of inflammatory and control lungs was 4.39 ± 0.64%ID/g and 2.85 ± 0.09%ID/g at 1 h p.i., and 5.46 ± 0.71%ID/g and 2.98 ± 0.49%ID/g at 6 h p.i., respectively. There was significant difference in the ^64^Cu^2+^ accumulation between the inflammatory group and control group (* *p* = 0.027, ^#^ *p* = 0.009). The mean ratio of the inflammatory lungs to the control was 1.54 ± 0.70 and 1.83 ± 0.55 at 1 and 6 h p.i., respectively (Figure 2C). For the group of mice with inflammatory lungs, the liver and kidney accumulation of radioactivity was 35.37 ± 5.13%ID/g and 19.53 ± 5.79%ID/g at 1 h p.i., and 28.55 ± 4.68%ID/g and 21.56 ± 3.58%ID/g at 6 h p.i., respectively. Moreover, the muscle uptake was 1.09 ± 0.05%ID/g and 0.95 ± 0.25%ID/g at 1 h and 6 h p.i., respectively (Figure 2B). 

### 2.3. Pathological Results

**Figure 3:**
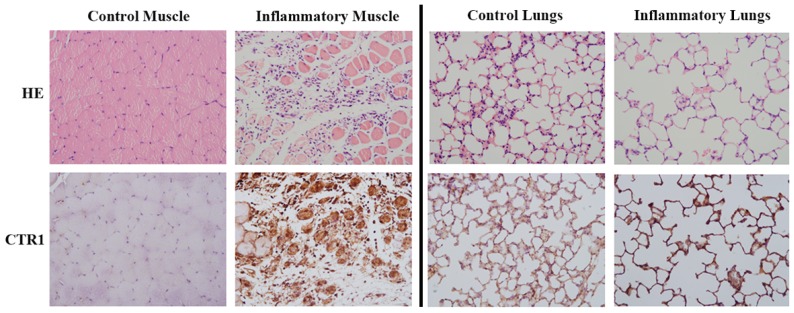
Microscopically, H&E and immunohistochemical CTR1 staining of the control muscle and inflammatory muscle (**Left**), and control lungs and inflammatory lungs (**Right**) (original magnification ×100).

As shown in Figure 3 left, hematoxylin and eosin (H&E, ×100) staining showed that muscle tissues were injured with infiltrating inflammatory cells after the induction of H_2_O_2_. Compared with almost no CTR1 expression of the control muscle, CTR1 levels were obviously increased in the inflammatory muscle based on immunohistochemical (IHC, ×100) staining, which were mainly presented in both injured muscle tissues and infiltrating inflammatory cells. The H&E image of Figure 3 right demonstrated that lung tissues were obviously injured with infiltrating inflammatory cells after the induction of LPS. Different from the control muscle, CTR1 was observed to be expressed in normal lung tissues, which was consistent with previous findings [21], but it was not as much as the inflammatory lung induced by LPS. 

### 2.4. Western Blot Analysis

**Figure 4:**
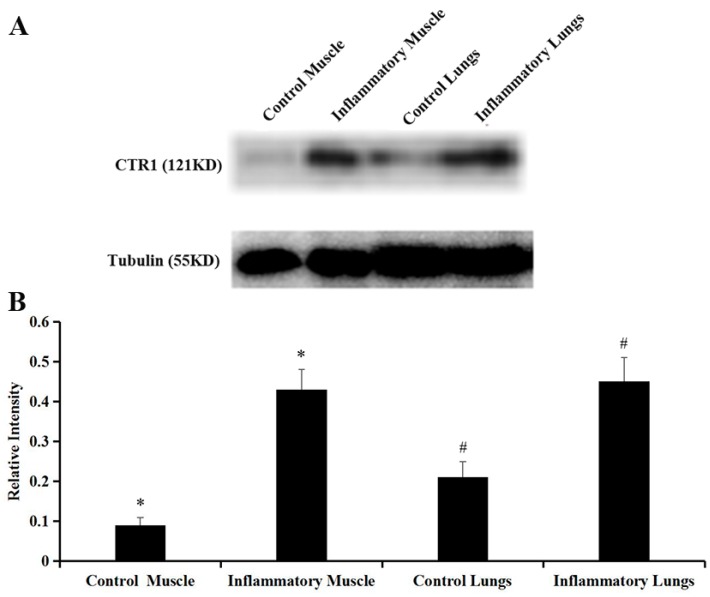
(**A**) Western blot analysis of CTR1 levels of the control and inflammatory muscle and lung tissues; (**B**) Quantification of the results of western blot analysis (* *p* = 0.000 control muscle vs. inflammatory muscle; ^#^ *p* = 0.003 control lungs vs. inflammatory lungs).

The levels of CTR1 expression in the inflammatory muscle tissues were significantly higher than that of the control group (0.43 ± 0.05 vs. 0.09 ± 0.02, * *p* = 0.000, Figure 4A,B). Similarly, compared with CTR1 expression in the control lung group (0.21 ± 0.04), increased CTR1 expression was observed in the inflammatory lung group (0.45 ± 0.06) (^#^ *p* = 0.003, Figure 4A,B).

## 3. Discussion

A variety of copper radionuclides (^64^Cu, ^67^Cu, etc.) have been used in the field of nuclear medicine, and they offer versatile choices for applications in radionuclide imaging and therapy. Particularly, ^64^Cu has an intermediate half-life of 12.7 h and unique decay profile (β+: 18%, β−: 38%, and electron capture: 44%), which make it a favorable option for radiolabeling nanoparticles, antibodies, antibody fragments, peptides, and small molecules for PET imaging and radionuclide therapy [22,23]. Furthermore, ^64^CuCl_2_ has been reported as a promising PET probe for imaging animal models with tumors through targeting of CTR1 [10,12,14,15]. More importantly, some studies have shown that ^64^CuCl_2_-PET/CT has been used in human study, which prove the quick translation of ^64^CuCl_2_ to the clinic over other tracers [16,24]. In this study, considering the previous publications that revealed the important role of Cu^2+^ and CTR1 in the pathogenesis of inflammation [18,19,20], we explore ^64^CuCl_2_ as a novel radiotracer for PET imaging of inflammation and further investigate the relationship between ^64^Cu^2+^ accumulation and CTR1 levels in inflammatory tissues in mice models.

Two representative inflammatory animal models have thus been selected for testing our hypothesis, which are muscle inflammation during the development of muscle injury after the local intramuscular injection of H_2_O_2_ and lung inflammation during the development of acute lung injury (ALI) after inhaled LPS. As expected, compared with the control muscle, the inflammatory muscle showed about four-fold elevated ^64^Cu^2+^ uptake based on the quantitative analysis of PET images. Consistent with PET finding, it is also notable that compared with the controls, CTR1 expression of the inflammatory muscle has been significantly increased. Therefore, our data clearly indicate that ^64^Cu^2+^ accumulation is associated with increased CTR1 expression, and ^64^CuCl_2_ can successfully image inflammatory muscle through CTR1 targeted PET imaging.

C57BL/6 mice develop a lung inflammatory response to inhaled LPS, which has been verified to closely replicate many physiological and biological features of human acute lung injury [25]. Kim ES et al. [21] reported that CTR1 expression was found in normal pulmonary epithelial tissues, which is similar to our study. Although CTR1 expression has been found in the normal lungs, approximately two-fold increased expression in the inflammatory lungs has been observed in our study. The results of PET images are consistent with IHC and western blot analysis. Our study clearly indicates that the degree of copper accumulation is correlative with CTR1 levels. The higher CTR1 levels, the more radioactive copper was uptaken. Therefore, ^64^CuCl_2_ may serve as a valuable tool to image the change of CTR1 expression of lung tissues after the induction of LPS.

Peng F et al. [26] and Xie F et al. [27] once evaluated ^64^CuCl_2_ uptake by muscular injury and traumatic brain injury with PET/CT, respectively. Their studies demonstrated that ^64^CuCl_2_ had potential to be a new radiotracer for the assessment of injury, but the molecular mechanism was not clarified. Moreover, Xie F et al. [27] showed that, compared with significantly increased ^64^Cu^2+^ uptake by the electroporation-injured muscular tissue, only minimal increase ^64^Cu^2+^ uptake by the muscle with LPS-induced inflammation. However, in the present study, IHC result showed CTR1 was highly expressed in both injured muscle tissues and infiltrating inflammatory cells. ^64^Cu^2+^ accumulation is associated with increased CTR1 expression, therefore ^64^CuCl_2_ should be uptaken by both injured muscle and inflammatory cells. Moreover, consistent with previous studies, our work also shows that ^64^CuCl_2_ displays high accumulation in the liver and kidney, which suggests that ^64^CuCl_2_ is mainly metabolized through hepatobiliary and kidney systems. These results are consistent with previous findings that ^64^Cu can bind with superoxide disumutase, which is widely distributed in the cytosol of eukaryotic cells and abundant in the liver and kidney [28].

Inflammation imaging has been challenging over the past decades, and the quest continues to find an ideal imaging agent. Recent studies reported that ^68^Ga-Citrate could be as a possible agent for PET imaging of inflammation based on the transferrin receptor. Radionuclide ^68^Ga is good for early time imaging, generator-based, and easy for clinical use. However, high background activity of ^68^Ga-Citrate in the thorax and upper abdomen may interfere with detecting lesions in these regions. Moreover, the half-life of ^68^Ga is short (68 min), which may be advantageous from low dosimetry to the patients, but disadvantageous for longer periods of study [29]. 

With the expected growth of PET/CT examination to be an essential strategy in clinical cancer and other benign diseases management, there is a recognized need for new PET probes to address clinical challenges. In this endeavor, CTR1 becomes a new target of inflammation imaging, and ^64^Cu has an intermediate half-life of 12.7 h, and the need for imaging for longer periods could be warranted. The high background of ^64^Cu focuses on the upper abdomen, which shows relatively smaller field than ^68^Ga-Citrate. The prospect of using ^64^Cu in the form of simple Cu^2+^ ions as a PET probe is not only a cost-effective proposition but also seems poised to broaden the palette of molecular imaging probes in the foreseeable future [30]. Therefore, based on our study, ^64^CuCl_2_ shows high potential to be used as not only cancers but also a novel PET inflammation imaging probe through targeting CTR1 expression of inflammatory diseases. 

## 4. Materials and Methods

### 4.1. General

^64^CuCl_2_ was purchased from the Department of Medical Physics, University of Wisconsin at Madison (Madison, WI, USA). The pH was adjusted to 7.0 and ^64^CuCl_2_ solution was diluted with phosphate-buffered saline (PBS, 0.01 M, pH 7.4) buffer, which was obtained from Gibco/Invitrogen (Carlsbad, CA, USA). All other chemicals were purchased from Sigma unless otherwise specified. 

### 4.2. Animal Models of H_2_O_2_ Induced Muscle Inflammation

All procedures were approved by the Administrative Panel on Laboratory Animal Care (APALC 9547) at Stanford University. The six-to-seven-week-old male BALB/C mice (Charles River Laboratories, Boston, MA, USA) were anesthetized by isoflurane inhalation, and 20 μM H_2_O_2_ was then administered intra-muscularly into the right hind leg of the mice. The contralateral muscle of left hind leg which received no treatment was used as the control. Three days after the induction of inflammation by H_2_O_2_, the animal models were used for small animal PET/computed tomography (CT) imaging study. 

### 4.3. Animal Models of LPS Induced Lung Inflammation

The six-to-seven-week-old male C57BL/6 mice (Charles River Laboratories, Boston, MA, USA) were anesthetized by isoflurane inhalation, and 50 μg of LPS (from *Escherichia coli* serotype 0127: B8, Sigma, St. Louis, MO, USA) in 20 μL of saline (0.9% NaCl) was then administered via intranasal instillation [25]. The control group was consisted of normal mice. Three days after LPS administration, the mice were imaged by small animal PET.

### 4.4. Small Animal PET/CT Imaging of Inflammatory Muscle

PET/CT imaging of animal models of H_2_O_2_ induced muscle inflammation was performed using a small animal PET/CT scanner (Siemens Inveon). Mice (*n* = 4 for each group) were injected with approximately 3.7–5.55 MBq (100–150 μCi) of ^64^CuCl_2_ via the tail vein. At 1 and 6 h p.i., mice were anesthetized with 2% isoflurane (5% isoflurane for induction and 2% for maintenance in 100% O_2_) for imaging experiments. The images were reconstructed with two-dimensional ordered-subset expectation maximization algorithm with CT-based attenuation correction. Image files were analyzed using the vendor-supplied software Inveon Research Workspace (Preclinical Solutions, Siemens Healthcare Molecular Imaging). For each small-animal PET scan, three-dimensional (3D) regions of interest (ROIs) were drawn over the organs and tissues on decay-corrected whole-body images. The average radioactivity concentration in the ROI was obtained from the mean pixel values within the ROI volume. These data were converted to counts per milliliter per minute by using a predetermined conversion factor. The results were divided by the injected dose to obtain an image ROI derived percent injected dose per gram of tissue [14].

### 4.5. Small Animal PET Imaging of Inflammatory Lungs

Similar to PET scan of animal models of H_2_O_2_ induced muscle inflammation, animal models of LPS induced lung inflammation and the controls (*n* = 4 for each group) were also injected with approximately 3.7–5.55 MBq (100–150 μCi) of ^64^CuCl_2_ through the tail vein. The mice were then anesthetized and imaged at 1 and 6 h p.i., respectively.

### 4.6. Pathology

The mice were sacrificed after imaging studies, and muscle and lung samples (with or without inflammation) were obtained. The slides of paraffin-embedded muscle and lung samples were used for common H&E staining. To determine whether ^64^Cu^2+^ accumulation in the inflammatory muscle and lungs was associated with the levels of CTR1 expression, IHC staining was performed. The antibody for CTR1 (Novus Biologicals, Littleton, CO, USA) was diluted at 1:500 and incubated on tissue sections overnight at 4 °C.

### 4.7. Western Blot for CTR1 Levels

Proteins from muscle and lung tissues were resolved by sodium dodecyl sulfate polyacrylamide gel electrophoresis (SDS-PAGE). Proteins were transferred (400 mA, 1 h) in a Mighty Small Transphor Transfer Tank (R&D System) to activated polyvinylidene flouride membranes in buffer containing 20% (volume:volume) methanol. For detection of proteins, membranes were exposed to antibodies diluted in 1:1000 dilution: rabbit anti-Tubulin (Abcam, Cambridge, MA, USA); rabbit anti-CTR1 (Abcam, Cambridge, MA, USA) at 4 °C overnight. The secondary antibody, peroxidase linked anti-rabbit IgG (Jackson Immunochemicals Inc., West Grove, PA, USA) was applied for 1 h at room temperature. The immune complexes were then detected with Tanon 5200 (China), using Pierce ECL Western Blotting Substrate (Thermo Fisher, Waltham, MA, USA) according to the manufacturer’s instructions.

### 4.8. Statistical Analysis

The quantitative data were expressed as mean ± SD. Means were compared using the Student’s *t*-test. A 95% confidence level was chosen to determine the significance between groups, with *p* values of less than 0.05 indicating significant differences.
